# The *Q^c5^* Allele Increases Wheat Bread-Making Quality by Regulating SPA and SPR

**DOI:** 10.3390/ijms23147581

**Published:** 2022-07-08

**Authors:** Zhenru Guo, Qing Chen, Jing Zhu, Yan Wang, Yang Li, Qingcheng Li, Kan Zhao, Yue Li, Rui Tang, Xiaoli Shi, Kenan Tan, Li Kong, Yunfeng Jiang, Qiantao Jiang, Jirui Wang, Guoyue Chen, Yuming Wei, Youliang Zheng, Pengfei Qi

**Affiliations:** 1 State Key Laboratory of Crop Gene Exploration and Utilization in Southwest China, Sichuan Agricultural University, Chengdu 611130, China; guozhenru@stu.sicau.edu.cn; 2 Triticeae Research Institute, Sichuan Agricultural University, Chengdu 611130, China; qingchen83@sicau.edu.cn (Q.C.); zhujing@stu.sicau.edu.cn (J.Z.); wangyan6@stu.sicau.edu.cn (Y.W.); liyang1@stu.sicau.edu.cn (Y.L.); liqingcheng@stu.sicau.edu.cn (Q.L.); zhaokan6137@163.com (K.Z.); 15397768229@163.com (Y.L.); 15691269656@163.com (R.T.); 18790196562@163.com (X.S.); 2020312061@stu.sicau.edu.cn (K.T.); kongli@sicau.edu.cn (L.K.); jiangyunfeng@sicau.edu.cn (Y.J.); qiantaojiang@sicau.edu.cn (Q.J.); jirui.wang@gmail.com (J.W.); gychen@sicau.edu.cn (G.C.); ymwei@sicau.edu.cn (Y.W.)

**Keywords:** breeding, seed storage proteins, mRNA secondary structure, microRNA, processing quality

## Abstract

Common wheat (*Triticum aestivum* L.) is an important food crop with a unique processing quality. The *Q* gene positively regulates the processing quality of wheat, but the underlying mechanism remains unclear. Here, a new *Q* allele (*Q^c5^*) responsible for compact spikes and good bread performance was identified. Compared with the *Q* allele widely distributed in modern common wheat cultivars, *Q^c5^* had a missense mutation outside the miRNA172-binding site. This missense mutation led to a more compact messenger RNA (mRNA) secondary structure around the miRNA172-binding region, resulting in increased *Q^c5^* expression during the spike development stage and a consequent increase in spike density. Furthermore, this missense mutation weakened the physical interaction between Q^c5^ and storage protein activator (SPA) in seeds and suppressed the expression of *storage protein repressor* (*SPR*). These changes increased the grain protein content and improved the bread-making quality of wheat. In conclusion, a missense mutation increases *Q* expression because of the resulting highly folded mRNA secondary structure around the miRNA172-binding site. Furthermore, this mutation improves the bread-making quality of wheat by repressing the expression of *SPR* and influencing the physical interaction between Q and SPA. These findings provide new insights into the miRNA172-directed regulation of gene expression, with implications for wheat breeding.

## 1. Introduction

Common wheat (*Triticum aestivum*; 2n = 6x = 42, AABBDD) originated about 8000 years ago in the Fertile Crescent of the Middle East [1,2] and has since spread worldwide to become one of the most important food crops. Common wheat resulted from the hybridization between the domesticated tetraploid wheat *Triticum turgidum* (2n = 4x = 28, AABB) and the diploid *Aegilops tauschii* (2n = 2x = 14, DD) [2]. The Food and Agriculture Organization of the United Nations estimates that wheat provides approximately 20% of the calories and 22% of the proteins consumed by humans worldwide [3].

The unique processing quality of wheat is due to its seed storage proteins (SSPs), including gliadins and glutenins (i.e., gluten) [4]. Gliadins, which are monomeric compounds, can be classified as α- (α/β-), γ-, and ω-gliadins [5]. Glutenins consist of high molecular weight glutenin subunits (HMW-GS) and low molecular weight glutenin subunits (LMW-GS) [6], which form large macropolymers during the grain desiccation stage [7]. Glutenins strengthen wheat dough by conferring elasticity, whereas gliadins affect dough viscosity by conferring extensibility [8]. The SSP content (grain protein content; GPC) and composition are critical factors influencing the processing quality of wheat, and thus affect wheat price. Therefore, elucidating the mechanisms mediating SSP synthesis is necessary.

The synthesis of SSPs during the grain filling stage is primarily regulated by a network of *cis*-elements and their interacting transcription factors (TFs) [9,10,11]. Previous studies revealed that the accumulation of SSPs is controlled by several TF families, namely basic leucine zipper (bZIP), DNA binding with one finger (DOF), myeloblastosis (MYB), B3 DNA-binding domain transcription factor (B3), and no apical meristem (NAM)/ATAF subfamily/cup-shaped cotyledon (CUC) family transcription factor (NAC). The bipartite endosperm box consists of a GCN4-like motif (GLM; 5′-ATGAG/CTCAT-3′) and a prolamin box (P-box; 5′-TGTAAAG-3′). Earlier research indicated that GLM is recognized by bZIP TFs, such as BLZ1 and BLZ2 in barley [12,13] or storage protein activator (SPA) in wheat [14], whereas P-box is a binding site for DOF TFs, including prolamin box-binding factor (PBF) and scutellum and aleurone-expressed DOF (SAD) [12,15,16]. The RY repeat (5′-CATGCATG-3′), which is also a conserved motif in *SSP* gene promoters, is recognized by the B3 protein FUSCA3 [17,18,19]. Moreover, TaGAMyb regulates glutenin gene expression by directly binding to a 5′-C/TAACAAA/C-3′-like motif in the promoter of HMW-GS genes and by recruiting GCN5 to modulate histone acetylation [20]. Additionally, the MYB TFs TuODORANT1 and TaODORANT1 are suppressors of SSP synthesis [21]. An NAC family TF (i.e., storage protein repressor; SPR) binds to the *cis*-element 5′-CANNTG-3′ to suppress the accumulation of SSPs [22]. Another endosperm-specific NAC TF (TaNAC019) directly activates the expression of HMW-GS genes by binding to their promoters and by interacting with TaGAMyb [23]. Opaque-2, which is an endosperm-specific bZIP family member in maize, binds to the promoter of the gene encoding the 22 kD zein protein to activate transcription [24]. In wheat, SPA is an opaque-2-like protein, which activates the transcription of LMW-GS genes by binding to two GLMs [14]; furthermore, SPA also enhances the expression of HMW-GS genes by binding to two GLMs and G-box in their promoters [25,26]. Moreover, SPA can activate the expression of ω-gliadin genes [27]. In addition to binding to DNA, TFs may interact with each other (i.e., protein–protein interactions) to form relatively large TF complexes that regulate the expression of *SSP* genes [28]. Furthermore, the SPA heterodimerizing protein (SHP) represses HMW-GS and LMW-GS gene promoter activities [26].

MicroRNAs (miRNAs) comprise approximately 22 nucleotides and inhibit gene expression mainly by eliminating DNA, cleaving mRNA, and repressing translation [29]. In plants, miRNA172 targets the transcripts of *APETALA2* (*AP_2_*) [30,31] and a few *AP_2_*-*like* genes [32,33,34]. In wheat, the miRNA172–*AP_2_*-*like* system is crucial for regulating spike morphology [35,36].

The *Q* gene encodes an AP_2_ TF that regulates spike morphology along with miRNA172. This gene is located on chromosome 5AL and contains two AP_2_-binding domains as well as a miRNA172-binding site in the 10th exon [37,38]. Additionally, *Q* was critical for wheat domestication [37] and de-domestication [39]. A previous study demonstrated that four overexpressed *Q* alleles (*Q^c1^*–*Q^c4^*) with point mutations in their miRNA172-binding site are associated with increases in spike density, GPC, and wheat bread-making quality [40]. However, the underlying molecular mechanisms remain unclear. 

In this study, another wheat mutant with increased spike density and improved bread-making quality was identified. We demonstrated that a missense mutation flanking the miRNA172-binding site of the *Q* gene (*Q^c5^* allele) is responsible for the mutant phenotype. How *Q^c5^* increases the spike density and enhances the bread-making quality of wheat was also investigated.

## 2. Results

### 2.1. Phenotype and Segregation Analyses

**Figure 1:**
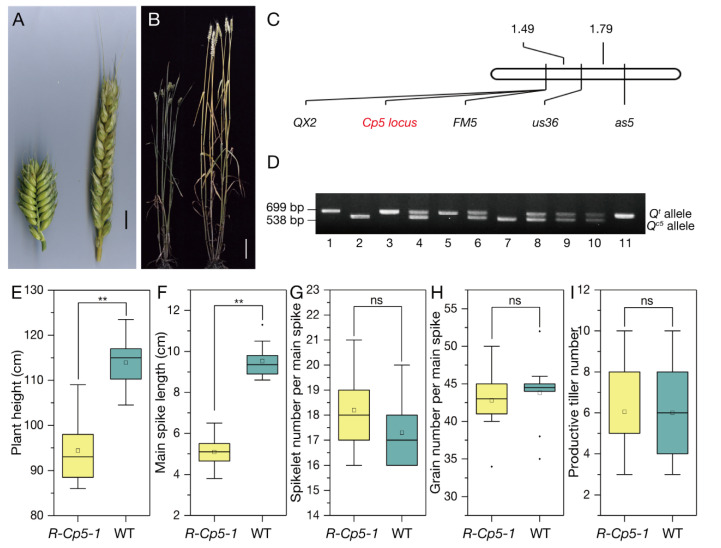
The mutant phenotype is controlled by the *Cp5* locus. (**A**) Spikes of *R-Cp5-1* (left) and the wild-type (WT) control (right). Scale bar, 1 cm. (**B**) *R-Cp5-1* (left) and WT (right) plants. Scale bar, 10 cm. (**C**) Segregation analysis of the *Cp5* locus. (**D**) Polymorphic molecular marker between *Q^t^* and *Q^c5^*, which co-segregated with the compact spike trait. Lane 1: QZ96; Lane 2: *R-Cp5-1*; Lanes 3–11: representative F_3_ individuals from *R-Cp5-1*/QZ96. (**E**–**I**) Comparison between *R-Cp5-1* and WT in terms of the plant height, spike length, spikelet number per spike, grain number per spike, and tiller number (*n* = 20; **, *p* ≤ 0.01; ns, not significant).

Compared with wild type (WT) wheat, the common wheat mutant *R-Cp5-1*, which was identified in a “Roblin” mutant population, was shorter in plant height and had a higher spike density (Figure 1A,B,E,F). There was no significant difference between *R-Cp5-1* and WT plants regarding spikelet number per spike, grain number per spike, and tiller number (Figure 1G–I). Using the compact spike as the target trait, the 160 F_2_ plants were segregated in the expected 3:1 ratio (χ^2^ = 0.533; *p* = 0.47), suggesting that the mutant phenotype was controlled by a single dominant locus (*Cp5*).

The *R-Cp5-1* mutant is phenotypically similar to the *S-Cp1-1* mutant, which carries the overexpressed *Q^c1^* allele with a point mutation in the miRNA172-binding site [40]. Therefore, we cloned the *Q* alleles in *R-Cp5-1* and “Roblin”. Unexpectedly, there were no variations in the miRNA172-binding region between the *R-Cp5-1* and “Roblin” *Q* sequences (Figure S1). The available molecular markers around *Q* [39] were used to determine whether the *R-Cp5-1* mutant phenotype is associated with *Q*. According to the linkage map, the markers *FM5* and *QX2* were closely linked with *Cp5* in the F_2_ population (Figure 1C). Additionally, *QX2* co-segregated with *Cp5* in the F_3_ population consisting of 1101 individuals (Figure 1D). Notably, *QX2* is an intragenic molecular marker of *Q* [39]. These results implied that *Q* is most likely responsible for the phenotype associated with the *Cp5* locus.

### 2.2. Gene Cloning and Expression Analysis

**Figure 2:**
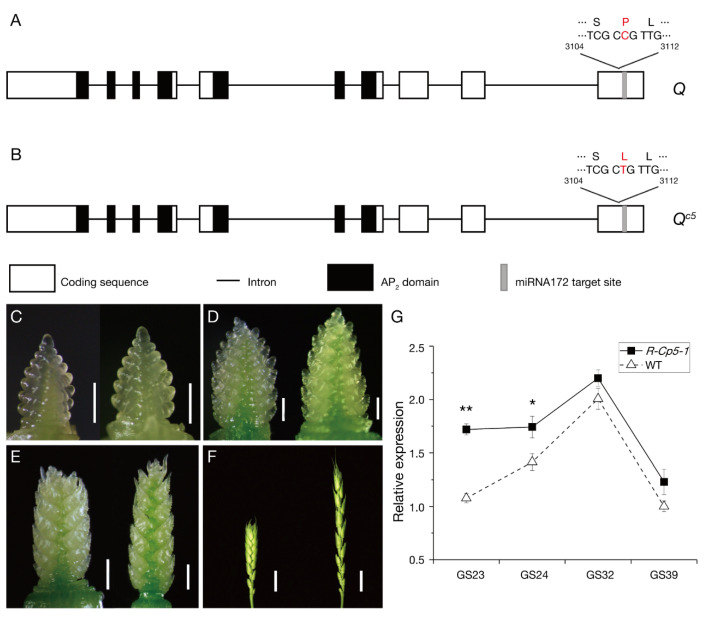
One missense mutation flanking the miRNA172-binding site increased the spike density and the *Q^c5^* expression level. (**A**) and (**B**) Schematic diagrams of the *Q* and *Q^c5^* alleles. A missense mutation (C in “Roblin” and T in *R-Cp5-1*) changes the 408th residue from proline (P) to leucine (L). (**C**–**F**) *R-Cp5-1* (left) and WT (right) spikes at GS23 (**C**), GS24 (**D**), GS32 (**E**), and GS39 (**F**). Scale bars, 0.05 cm in (**C**,**D**), 0.1 cm in (**E**), and 1 cm in (**F**). (**G**) Comparison of the *Q* and *Q^c5^* transcription levels in spikes at GS23, GS24, GS32, and GS39. Data are presented as the mean ± standard deviation (*n* = 3; *, *p* ≤ 0.05; **, *p* ≤ 0.01).

The full-length cDNA and genomic DNA sequences of *Q* in *R-Cp5-1* (GenBank No. MW419115) and “Roblin” (KX620763.1) were cloned and sequenced; then, sequence alignment revealed a missense mutation (C–T; Figure 2A,B; Figure S1) flanking the miRNA172-binding region. The new *Q* allele was named *Q^c5^*, which was added to the four reported *Q^c^* alleles (*Q^c1^*–*Q^c4^*; Figure S2) [40].

A qRT-PCR analysis was performed to assess whether this missense mutation increases the *Q* expression level. The *Q^c5^* and *Q* allele expression patterns in the mutant and the WT control, respectively, were similar, but *Q^c5^* was consistently more highly expressed (significantly higher at Growth Stage 23 (GS23) and GS24 [41]) than the *Q* allele in spikes at four growth stages (Figure 2C–G).

To clarify how the missense mutation enhances *Q^c5^* expression, the *Q* and *Q^c5^* mRNA secondary structures were compared. Unlike the corresponding region in *Q*, the miRNA172-binding region of *Q^c5^* was highly folded (Figure S3), which inhibited the binding of miRNA172.

### 2.3. Protein Structure Analysis

The secondary and tertiary (i.e., 3D) structures of the Q and Q^c5^ proteins were predicted to assess the biological significance of the amino acid substitution. The predicted structures indicated that compared with Q, Q^c5^ had more amino acid residues that can form an α-helix in the region around the changed residue (Figure S4).

### 2.4. Processing Quality Analysis

**Figure 3:**
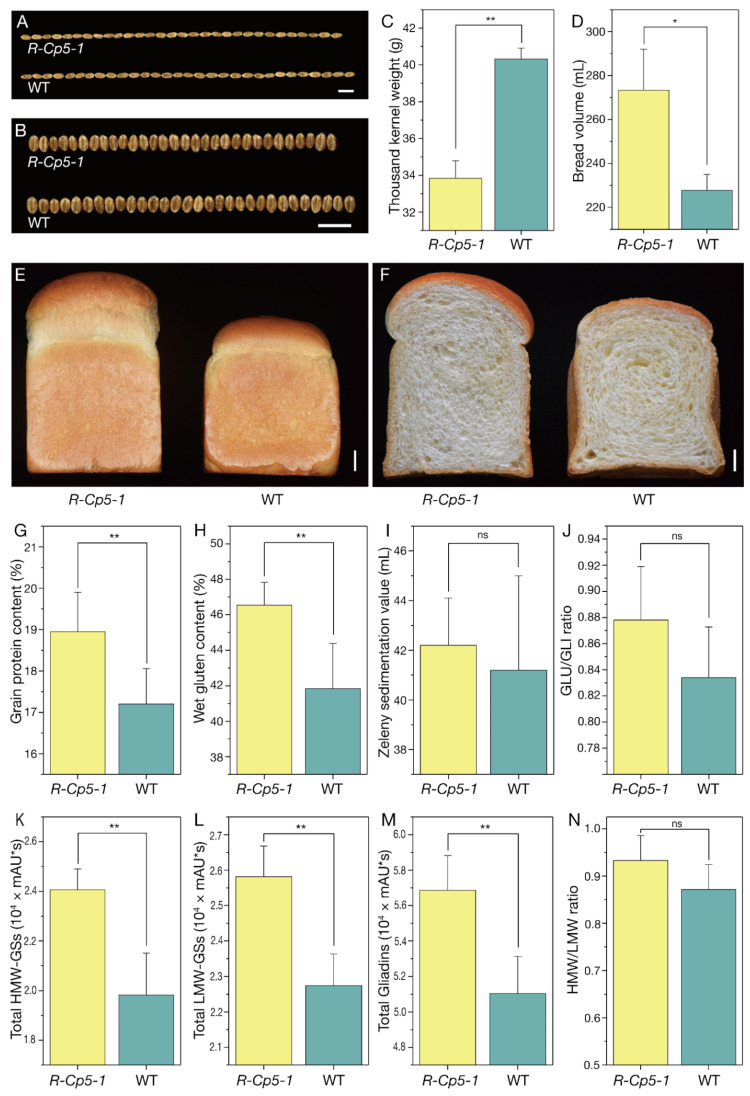
*Q^c5^* results in relatively small seeds and increased processing quality. Length (**A**) and width (**B**) of 30 seeds from *R-Cp5-1* (upper) and WT (lower). Scale bar, 1 cm. (**C**) Thousand kernel weight of *R-Cp5-1* and WT. (**D**–**F**) Comparison of bread made from *R-Cp5-1* and WT wheat. Scale bar, 1 cm. (**G**–**I**) Comparison of the grain protein content (**G**), wet gluten content (**H**), and Zeleny sedimentation value (**I**) between *R-Cp5-1* and WT. (**J**–**N**) The Glu/Gli ratio (**J**), total HMW-GS (**K**), total LMW-GS (**L**), total gliadins (**M**), and HMW/LMW ratio (**N**) were greater for *R-Cp5-1* than for WT. Data are presented as the mean ± standard deviation (*n* = 4; *, *p* ≤ 0.05; **, *p* ≤ 0.01).

To determine whether *Q^c5^* affects the wheat processing quality, the quality-related parameters of *R-Cp5-1* and WT were compared (Figure 3D–N). The GPC, wet gluten content, and Zeleny sedimentation value were higher for *R-Cp5-1* than for WT (Figure 3G–I). Additionally, the average bread volume was 20% greater for *R-Cp5-1* than for WT (Figure 3D–F). The glasshouse experiment also showed that GPC was significantly higher for *R-Cp5-1* than for WT (Figure S5). The RP-HPLC analysis indicated that the Glu/Gli and HMW/LMW ratios as well as the total HMW-GS, total LMW-GS, and total gliadin contents were greater for *R-Cp5-1* than for WT (Figure 3J–N; Figure S6). In contrast to its positive effect on processing quality, *Q^c5^* negatively affected grain size (Figure 3A,B) and thousand kernel weight (TKW) (Figure 3C).

### 2.5. Mechanisms Underlying the Effect of Q^c5^ on Processing Quality

The starch granule (SG) and protein body (PB) morphological characteristics in the mature seeds of *R-Cp5-1* and WT were examined using a scanning electron microscope. Compared with the WT samples, the PBs and SGs in three representative areas of *R-Cp5-1* were more tightly arranged, with fewer and smaller gaps. Furthermore, the SGs in WT were relatively exposed, whereas the SGs in *R-Cp5-1* were more tightly packed with PBs (Figure S7).

**Figure 4:**
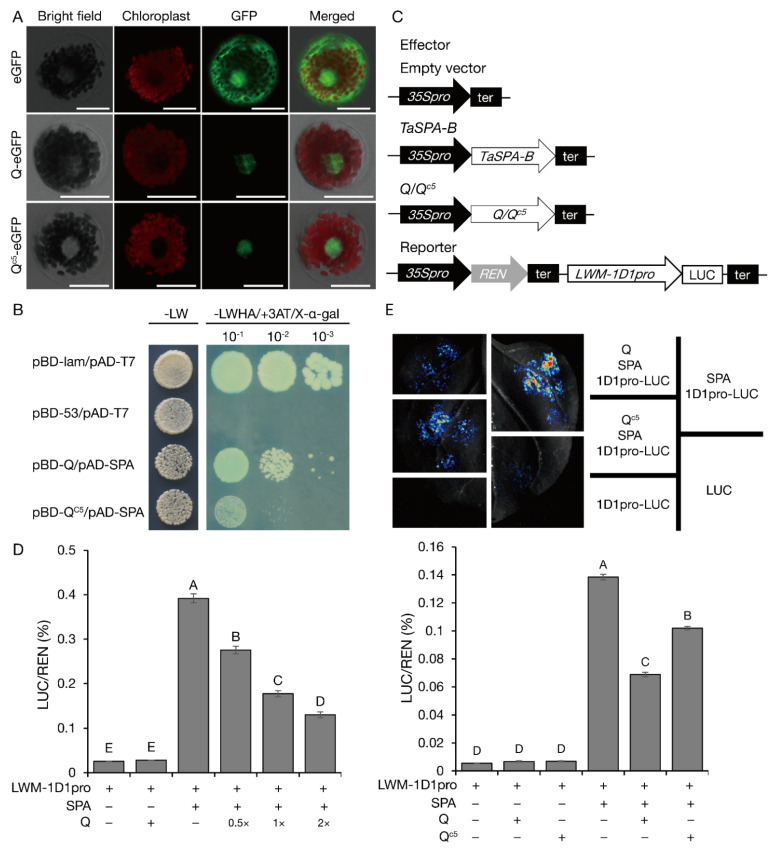
Q physically interacts with SPA to regulate the expression of *SSP* genes. (**A**) Q and Q^c5^ are localized to the nucleus of wheat mesophyll protoplasts. (**B**) Q^c5^ interacts with SPA more weakly than Q in yeast two-hybrid assays. Y2HGold yeast cells transformed with the labeled constructs were assayed for growth on SD medium lacking LW and for LacZ activities on SD-LWHA medium supplemented with X-α-gal. pAD, GAL4 activation domain; pBD, GAL4 DNA-binding domain; 4 mM 3AT was added to the SD-LWHA medium to repress self-activation. The pGBKT7 and pGADT7 empty vectors were used as the negative control, whereas the interaction between p53 and Lam served as the positive control. (**C**–**E**) Ability of Q/Q^c5^ and SPA to activate the LMW-GS gene promoter revealed by dual-luciferase transcriptional activity assays. (**C**) Schematic diagrams of the effector and reporter plasmids. *35S:SPA*, *35S:Q*, and *35S:Q^c5^* were constructed as effectors. The promoter of the LMW-GS gene *LMW-1D1* was fused to *LUC* to generate the reporter *LMW-1D1pro:LUC*. LUC, firefly luciferase; 35Spro, CaMV 35S promoter; REN, *Renilla reniformis* luciferase; ter, terminator. (**D**) Q inhibits the ability of SPA to activate the *LMW-1D1* promoter. (**E**) Q^c5^ + SPA activates the promoter more than Q + SPA. Representative leaves are shown in the upper panel, whereas the relative luciferase activities are presented in the lower panel. LUC/REN indicates the LUC-to-REN activity ratio. Data are presented as the mean ± standard deviation (*n* = 3). Capital letters above each column indicate significant differences at *p* ≤ 0.01. + and − respectively indicate the presence and absence of the indicated effector/reporter constructs, whereas × indicates the Q concentration.

**Figure 5:**
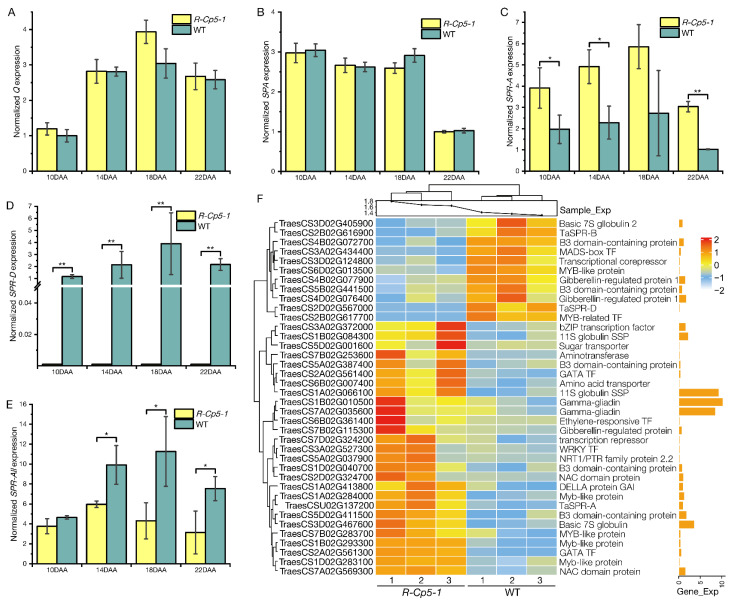
Relative expression of *Q* (**A**), *SPA* (**B**), *SPR-A* (**C**), *SPR-D* (**D**), and *SPR-All* (**E**) in developing seeds. Data are presented as the mean ± standard deviation (*n* = 3; *, *p* ≤ 0.05; **, *p* ≤ 0.01). (**F**) Heat map presenting the mean normalized expression of selected DEGs. The scale bar indicates the mean normalized expression level. Sample_Exp is the mean expression of all genes in every sample; Gene_Exp is the mean expression of each gene. The histograms reflect the relative expression levels. All DEGs are listed in Tables S5 and S6.

In the subcellular localization experiment involving wheat mesophyll protoplasts, Q^c5^ and Q were distributed in the nucleus (Figure 4A). However, Q cannot directly activate the transcription of *SSP* genes (Figure S8). Therefore, we speculated that Q indirectly regulates the expression of *SSP* genes by interacting with other TFs. Hence, a yeast two-hybrid assay was performed to identify the TFs encoded in a cDNA library that can interact with Q. Among the six TFs that were obtained (Table S1), SPA encoded by TraesCS1B02G343500 was the most likely interacting partner of Q. Previous research demonstrated that SPA directly regulates the expression of *SSP* genes [14,25,26]. Accordingly, SPA was selected for further research and was verified to interact with Q. The *Q^c5^* expression level was slightly, but not significantly, higher than the *Q* expression level in developing seeds, but there was no correlation between the *SPA* and *Q* expression patterns (Figure 5A,B), suggesting that *Q* does not regulate *SPA* expression. Notably, the interaction between Q^c5^ and SPA was weaker than that between Q and SPA (Figure 4B).

Because SPA is reportedly an activator of LMW-GS gene expression [14], dual-luciferase reporter assays were performed to determine whether Q and SPA can regulate *LMW-1D1* expression (Figure 4C–E). As expected, SPA significantly increased the LUC activity, whereas Q and Q^c5^ had no effect. However, compared with the effect of SPA alone, the co-infiltration of Q/Q^c5^ and SPA resulted in decreased LUC activity. The extent of this decrease was positively associated with the abundance of Q (i.e., dosage effect) (Figure 4D). The inhibitory effect of Q^c5^ was weaker than that of Q (Figure 4E) because of the weaker physical interaction between Q^c5^ and SPA than between Q and SPA (Figure 4B). Overall, the activation of LMW-GS gene expression by SPA can be inhibited by Q, but a mutation from Q to Q^c5^ weakens this inhibition.

### 2.6. Effect of Q^c5^ on the Seed Transcriptome

To further clarify how *Q^c5^* improves the bread-making quality of wheat, the transcriptomes of immature seeds at 14 days after anthesis (DAA) were compared between *R-Cp5-1* and WT (Table S2). A total of 597 DEGs were identified, including 391 up-regulated and 206 down-regulated genes in *R-Cp5-1* (Tables S3 and S4; Figure S9). The GO enrichment analysis revealed 31 significantly enriched terms associated with *Q*^c5^ (Table S5; Figure S10). A total of 90 KEGG pathways were assigned to 108 up-regulated and 17 down-regulated genes (Table S6; Figure S10).

We searched for TFs that reportedly regulate *SSP* gene expression. Only *SPR* expression was significantly altered (Table 1), suggesting that *Q* regulates the expression of *SSP* genes by modulating *SPR* expression. The homologous alleles of *SPR* in the three sub-genomes were differentially regulated. Specifically, *SPR-B* (TraesCS2B02G616900) and *SPR-D* (TraesCS2D02G567000) expression levels were down-regulated in *R-Cp5-1*, which was in contrast to the up-regulated expression of *SPR-A* (TraesCSU02G137200) (Table 1; Figure 5C–F). Additionally, *SPR-D* was unexpressed in *R-Cp5-1* and *SPR-B* was expressed at very low levels in WT and *R-Cp5-1* (Table S4). The qRT-PCR analysis of *SPR* expression indicated that the total expression of all *SPR* alleles (*SPR-All*) decreased significantly in *R-Cp5-1* developing seeds (Figure 5C–E).

## 3. Discussion

Wheat domestication was a long process that was closely related to the development of human societies and the establishment of civilizations [42]. Additionally, *Q* is arguably the most important domestication gene in cultivated wheat. The *Q* allele was the result of a spontaneous mutation in the miRNA172-binding region of the *q* allele in wild wheat, which increased the *Q* expression level [37]. The *Q* allele confers the free-threshing characteristic of wheat and influences several domestication-related traits, including rachis fragility, glume toughness, spike architecture, flowering time, and plant height [37,38,43,44,45,46,47]. The *Q^c1^* allele originated from a point mutation within the miRNA172-binding region of the *Q* allele, which decreases the miRNA172-directed cleavage of *Q^c1^* transcripts, thereby leading to increased expression levels and enhanced wheat bread-making quality [40]. Therefore, point mutations in the miRNA172-binding site can increase the *Q* transcription level and influence multiple traits, including processing quality.

Processing quality is a major trait of interest among wheat breeders, but relatively few genetic loci with significant effects on processing quality and useful for wheat breeding have been reported. Joppa [48] detected a QTL explaining 66% of the variation in GPC. The identified gene in this QTL encodes a NAC TF that is associated with GPC increases of approximately 14 g kg^−1^, but it also accelerates senescence [49]. Gao [23] identified an elite *TaNAC019* allele related to bread-making quality. Compared with the effects of the *Q* allele common among modern wheat cultivars, we demonstrated that *Q^c1^* is correlated with a GPC increase of about 60 g kg^−1^ and a loaf volume increase of 37%. In this study, we analyzed the *Q^c5^* allele to confirm *Q* affects GPC (Figure 3G; Figure S5) and loaf volume (Figure 3D–F). Unfortunately, *Q^c1^* is inappropriate for breeding because it leads to decreased grain yields, extreme dwarfism, and the production of compact spikes [40]. A previous study identified *Q^c1^-N8* as a new allele derived from *Q^c1^*, with a missense mutation in the sequence encoding the second AP_2_ domain [50]. This new allele reverses the unfavorable agronomic characteristics resulting from *Q^c1^* while also positively affecting GPC and grain yields [50]. Wheat is an important protein source for humans [3]. Therefore, the influence of *Q* on GPC suggests that *Q* should be targeted by wheat breeding programs.

The *Q^c5^* allele improves the bread-making quality of wheat by enhancing the accumulation of SSPs and by increasing the Glu/Gli ratio (Figure 3J). The unique properties of wheat depend on its SSPs [4]. An increase in the SSP contents in mutant grains inevitably leads to the production of more PBs, which combine to form a matrix protein that binds to SGs during the seed dehydration stage (Figure S7).

The value of *Q* is associated with the number of point mutations in its miRNA172-binding site. The transition from *q* to *Q* (one point mutation in the miRNA172-binding site) during wheat domestication had a profound effect on the grain yield and shape [51]. The stepwise mutation from the *Q* allele to the *Q^c1^-N8* allele (another point mutation in the miRNA172-binding site) further increased the grain yield by increasing TKW and the grain number per spike [50]. In plants, a high sequence complementarity between a specific miRNA and its target mRNA is required for the miRNA–target interaction [52,53]. The cleavage efficiency of miRNAs can be modified by changes in their binding regions within target genes [52,54]. Several studies have focused on the interactions between miRNA172 and *AP2*-*like* genes in grass species [55,56,57,58]. In addition to sequence complementarity, another factor influencing miRNA regulatory effects is the mRNA secondary structure [59]. In this study, *Q^c5^*, which has a point mutation outside of the miRNA172-binding site, was more highly expressed than *Q* because the miRNA172-binding region is more highly folded in *Q^c5^* than in *Q* (Figure S3). This finding may form the basis of a new method for further increasing the *Q* expression level.

The C-terminal region of Q protein is related to grain size. Both *Q^c5^* and *Q^c1^* are transcribed at a higher level than the *Q* allele (Figure 2G) [40]. However, increased grain size is associated with *Q^c1^* (Figure S11), but not *Q^c5^* (Figure 3A,B). The missense mutation that changes a proline in Q to a leucine in Q^c5^ is outside the AASSGF box. Moreover, the amino acid change alters the protein structure, resulting in decreased protein activity. Proline is detrimental to the formation of α-helices [60,61], whereas leucine tends to be included in helical structures. Therefore, replacing the proline (amino acid 408) at the end of the α-helix in Q with leucine extends the α-helix in Q^c5^ by two residues. Specifically, the α-helix ends at amino acid residues 409 and 411 in Q and Q^c5^, respectively (Figure S4B–E). The missense mutations in the first four *Q^c^* alleles affect the conserved AASSGF box of Q (Figure S12), resulting in minor changes to the secondary structure of the AASSGF domain in the four Q^c^ proteins (Figures S13 and S14). Moreover, there were no differences in the 3D structures of Q and Q^c^. Therefore, the opposite effects of Q^c5^ and Q^c1^ on grain size may be due to the diversity in their protein structures.

**Figure 6:**
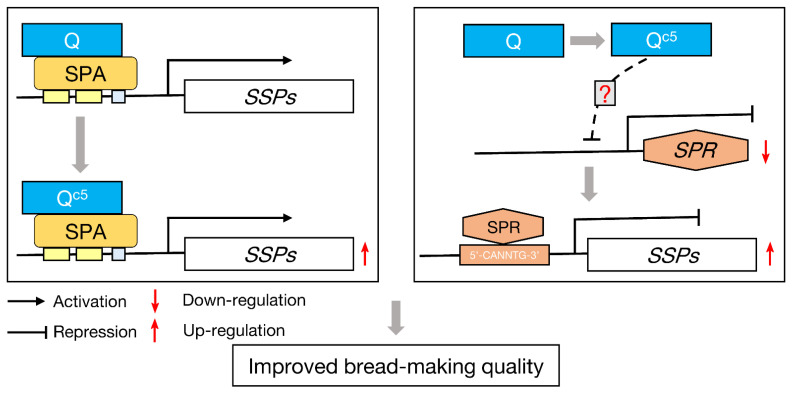
Schematic diagram of the regulatory effect of *Q^c5^* on *SSP* gene expression in wheat seeds. The activation of *SSP* expression by SPA is inhibited by Q, but the missense mutation from *Q* to *Q^c5^* weakens this inhibition, resulting in more SSP accumulation. Moreover, the missense mutation in *Q^c5^* down-regulates *SPR* expression; since *SPR* functions as a repressor of *SSP* genes, the suppression of *SPR* results in up-regulated *SSP* expression levels. Taken together, the up-regulated SSPs expression results in improved bread-making quality. Solid and dotted arrows indicate direct and indirect regulation, respectively. The question mark indicates the regulatory effect of *Q* on *SPR* expression remains to be elucidated.

The results of this study have clarified the mechanism underlying the effect of *Q^c5^* on wheat processing quality (Figure 6). Specifically, *Q^c5^* enhances *SSP* gene expression in two ways. First, SPA can activate the transcription of glutenin genes [14], but Q has an inhibitory effect on SPA (Figure 4E). The missense mutation in *Q^c5^* weakens the physical interaction between the encoded protein and SPA, leading to a weaker inhibitory effect on SPA and then increased *LMW-GS* gene expression (Figure 4D–E). Second, the missense mutation in *Q^c5^* is related to the down-regulated expression of *SPR*. However, our ChIP-seq analysis did not detect the enrichment of Q in the *SPR* region (unpublished data). Accordingly, how *Q* regulates *SPR* expression remains to be elucidated.

## 4. Materials and Methods

### 4.1. Plant Materials and Growth Conditions

Common wheat cultivar “Roblin” was treated with 0.6% ethyl methanesulfonate (Sigma-Aldrich, St Louis, MO, USA). A mutant (*R-Cp5-1*) with increased spike density and its corresponding wild-type (WT) control were isolated from a single M_7_ heterozygous plant. A total of 160 F_2_ individuals derived from the *R-Cp5-1* × QZ96 hybridization were used for the chi square (χ^2^) test and segregation analysis. An F_3_ population comprising 1101 individuals was generated from heterozygous F_2_ plants (*R-Cp5-1* × QZ96) and then used for the segregation analysis. Hexaploid wheat line QZ96 has speltoid-like spikes and contains the *Q^t^* allele (i.e., *Q* allele with a transposon insertion [39]). Plants were grown in a plot with a row spacing of 20 cm × 10 cm at the experimental farm of Sichuan Agricultural University, Chengdu, China. A nitrogen: phosphorous: potassium (15:15:15) compound fertilizer was applied (750 kg per hectare) before sowing.

A field experiment was conducted according to a randomized block design with five replicates during the 2017–2018 wheat growing seasons to compare the agronomic traits and processing parameters of *R-Cp5-1* and WT. Each replicate was planted in a 2 m × 4 m plot with a row spacing of 20 cm × 5 cm. At GS80 [41], 20 plants per line were randomly selected for an analysis of agronomic traits, including plant height (cm), main spike length (cm), spikelet number per main spike, grain number per main spike, and productive tiller number. The wheat materials were grown as described above.

One glasshouse (16 h day (23 °C)/8 h night (18 °C) cycle) experiment was conducted in 2019 to analyze gene expression in grains. Plants were grown in 2 L pots (four *R-Cp5-1* plants and four WT plants per pot) filled with a loam-based substrate (Pindstrup, Ryomgaard, Denmark). The pots were arranged according to a randomized complete block design with three replicates. Plants were watered as needed and fertilized before planting with a nitrogen: phosphorous: potassium (15:15:15) compound fertilizer.

*Nicotiana benthamiana* plants were grown in a glasshouse at 22 °C with a 16 h day/8 h night cycle. When plants had six leaves, the youngest leaves longer than 1 cm were infiltrated with *Agrobacterium tumefaciens* and maintained in the glasshouse for the duration of the dual-luciferase reporter assay.

### 4.2. DNA Extraction and Segregation Analysis

Genomic DNA was extracted from the young leaves of 160 F_2_ individuals and 1101 F_3_ individuals derived from the *R-Cp5-1* × QZ96 hybridization using cetyltrimethylammonium bromide [62]. The subsequent PCR amplifications were performed using the following program: 94 °C for 4 min; 35 cycles of 94 °C for 30 s, 55 °C for 30 s, and 72 °C for 30 s; 72 °C for 10 min. The PCR products were separated on 8% polyacrylamide gels or 1% agarose gels. Compact/normal spike was selected as the target trait for the segregation analysis. The spike morphology of all individuals was investigated at GS80.

Four molecular markers [39] that were polymorphic between the parents (*R-Cp5-1* and QZ96) were used for the segregation analysis; then, a linkage map was constructed using the MAP function of IciMapping 4.1 (Institute of Crop Science and CIMMYT China, CAAS, Beijing, China).

### 4.3. RNA Extraction and Gene Expression Analysis

Developing spikes were collected from the *R-Cp5-1* and WT plants grown for the field experiment at GS23, GS24, GS32, and GS39. Three biological replicates were collected, with at least 10 spikes per replicate. Developing grains were collected from the *R-Cp5-1* and WT plants grown for the glasshouse experiment at 10, 14, 18, and 22 DAA. Three biological replicates were collected per time-point. The collected samples were immediately frozen in liquid nitrogen and then stored at −80 °C. The plant samples were ground to a fine powder in liquid nitrogen. Total RNA was extracted from the ground material using the Plant RNA Extraction kit (Biofit, Chengdu, China) and then was quantized using the NanoDrop 8000 spectrophotometer (Thermo Scientific, Waltham, MA, USA). First-strand cDNA was synthesized from 1 μg total RNA using the PrimeScript™ II 1st Strand cDNA Synthesis Kit (Takara, Dalian, China).

All RNA samples extracted from spikes and seeds were used for qRT-PCR analysis by using the ChamQ™ Universal SYBR^®^ qPCR Master Mix (Vazyme, Nanjing, China). All experiments were performed according to the manufacturer’s instructions. Housekeeping genes, namely the genes encoding the scaffold-associated region DNA-binding protein (NCBI UniGene: *Ta.14126*) and methionine aminopeptidase 1 (*Ta.7894*) [63], were used for normalizing gene expression data. The qRT-PCR primers are listed in Table S7.

The RNA extracted from seeds at 14 DAA was used for a transcriptome analysis. Specifically, the RNA was sequenced using the Illumina NovaSeq platform at Tcuni Biotech (Chengdu, China). The RNA quantity and integrity were determined using the NanoDrop 8000 spectrophotometer (Thermo Scientific, Waltham, MA, USA) and the Agilent 4200 TapeStation (Agilent Technologies, Santa Clara, CA, USA). The RNA-seq libraries were constructed using the VAHTS^®^ Stranded mRNA-seq Library Prep Kit for Illumina. The libraries were amplified by PCR and quantified by qRT-PCR. The default parameters of Kallisto (v0.42.4) [64] were used for quantifying transcript abundances. The edgeR (v3.12.1) program was used to detect differentially expressed genes (DEGs) between *R-Cp5-1* and “Roblin” according to the following criteria: adjusted *p* < 0.05 and an absolute fold-change > 2.0 [65]. The GOseq (v1.22.0) program was used for a Gene Ontology (GO) enrichment analysis of the DEGs [66] on the basis of the Wallenius non-central hyper-geometric distribution, which can adjust for the gene length bias among DEGs. A Kyoto Encyclopedia of Genes and Genomes (KEGG) pathway enrichment analysis of the DEGs was completed using KOBAS (v2.0) [67]. The transcripts per kilobase of exon model per million mapped reads (TPM) value was used for calculating gene expression levels.

### 4.4. Gene Cloning

The cDNA and genomic DNA sequences of candidate genes in *R-Cp5-1* and “Roblin” were amplified by PCR. The *HMW-Bx7*, *HMW-Dx5*, *HMW-Dy10*, and *LMW-1D1* promoters in “Roblin” were also amplified. Phanta Max Super-Fidelity DNA Polymerase (Vazyme) was used for the PCR amplifications, which were conducted in a 50 μL mixture comprising genomic DNA or cDNA, 100 μΜ each dNTP, 4 pmol each primer, 1 U DNA polymerase, and 25 μL 2× buffer (with 4 mM Mg^2+^). The PCR amplifications were completed in the Mastercycler^®^ nexus programmable thermal cycler (Eppendorf, Wesseling-Berzdorf, Germany) using the following program: 95 °C for 3 min; 35 cycles of 95 °C for 15 s, 60 °C for 15 s, and 72 °C for 1–2 min; 72 °C for 10 min. The PCR products were separated on a 1.5% agarose gel. The expected fragments were purified and inserted into the pMD19-T vector (Takara). Positive colonies were verified by Sanger sequencing (Sangon, Chengdu, China). The cloning and sequencing experiments were repeated at least three times. The primers used are listed in Table S7.

### 4.5. Structure Prediction

The secondary structures of the mRNA sequences of *q* (GenBank No. AY702957.1), *Q* (KX620763.1), *Q^c5^* (MW419115), and another four overexpressed *Q* alleles (i.e., *Q^c1^*–*Q^c4^*, KX620765, KX620766, KX620767, and KX620768) [40] were predicted using the Vienna RNA fold server (http://rna.tbi.univie.ac.at/; accessed on 1 March 2018) [68,69]. The secondary structures around the miRNA172-binding site were clipped and compared. The secondary structures of the deduced peptides encoding by *q*, *Q*, *Q^c5^*, and *Q^c1^*–*Q^c4^* were predicted using the default parameters of Antheprot 6.9.3 [70].

The three-dimensional (3D) structures of *q*, *Q*, *Q^c5^*, and *Q^c1^*–*Q^c4^* were predicted using I-TASSER (http://zhanglab.ccmb.med.umich.edu/I-TASSER/; accessed on 10 August 2018) [71,72,73] as described by Zhao [74]. The full-length *Q* and *Q^c5^* structures were compared using TM-align (https://zhanggroup.org/TM-align/; accessed on 27 December 2018) as described by Zhang [75]. After simulating the substitution of the 408th residue (from proline in *Q* to leucine in *Q^c5^*), the resulting sequences were aligned using STRUM (https://zhanggroup.org/STRUM/; accessed on 1 January 2019) [76]. The *q*/*Q* and *Q*/*Q^c1^–Q^c4^* protein structures were also compared using TM-align. Moreover, the 3D protein structures were visualized using UCSF Chimera (version 1.13.1; http://www.rbvi.ucsf.edu/chimera/; accessed on 1 January 2019) as described by Pettersen [77].

### 4.6. Processing Quality Analysis

All plants grown in the experimental plot were harvested, threshed, and sun-dried at approximately 35 °C to a constant weight. Grain samples were stored at room temperature for 2 months before milling. Two grain portions (500 kernels) were randomly selected and weighed to calculate the TKW (the difference in kernel weight between the two portions was less than 5%). The grain moisture content was measured using the MJ33 moisture analyzer (Mettler Toledo, Switzerland) and adjusted to 16.5% before milling. Each grain sample (1 kg) was milled using the Chopin^®^ CD1 Mill (Chopin Technologies, Villeneuve-la-Garenne, France). The GPC (dry weight), Zeleny sedimentation value, and wet gluten content were determined as described by Wang [78].

A baking test was performed according to the AACC method 10.09-01 [40], with some modifications. Briefly, the standard rapid-mix-test with 35 g flour (14% moisture content) was used. The test was performed using three biological replicates, with two loaves of bread per flour sample. The bread volume was determined using the BVM6630 volume meter (Perten, Hägersten, Sweden).

### 4.7. Reversed-Phase High-Performance Liquid Chromatography (RP-HPLC)

An RP-HPLC analysis was performed to quantify the gliadin, HMW-GS, and LMW-GS contents and calculate the glutenins-to-gliadins (Glu/Gli) and HMW-GS-to-LMW-GS (HMW/LMW) ratios as described by Dupont [79] and Zheng [80], with some modifications. The total HMW-GS, LMW-GS, and gliadin contents were estimated by integrating the relevant RP-HPLC peaks in the chromatograms. The Glu/Gli ratio was calculated according to the relative contents.

### 4.8. Subcellular Localization

The full *Q* and *Q^c5^* coding sequences (CDSs) were cloned into the pCAMBIA2300-eGFP (enhanced green fluorescent protein) vector. The primers used are listed in Table S7. The resulting vectors were inserted into wheat mesophyll protoplasts. After a 16 h incubation, the transfected protoplasts were examined using the STELLARIS STED/EM CPD300 confocal microscope (Leica, Germany). Wheat mesophyll protoplasts were prepared and transfected as described by Brandt [81].

### 4.9. Yeast One- and Two-Hybrid Analyses

To screen for Q-interacting proteins, equal amounts of RNA extracted from grain samples at 5, 10, 15, 20, 25, and 30 DAA were combined for the construction of an AD-prey yeast library (pGADT7-Rec) (Oebiotech, Shanghai, China). The *Q* CDS was inserted into the pGBKT7 vector (Q-BD). Subsequently, Q-BD and the AD-prey library were used for an examination of protein–protein interactions according to the Matchmaker Gold Yeast Two-Hybrid Library screening user manual (Clontech, Saint-Germain-en-Laye, France).

To compare the physical interaction between Q/Q^c5^ and SPA (GenBank No. Y09013.1), the *SPA* CDS was cloned from the cDNA of 14 DAA grains and then incorporated into the prey vector pGADT7 (*AD-SPA*). The resulting vector and the *Q-BD* or *Q^c5^*-*BD* bait vector were used for the co-transformation of Y2H Gold yeast cells. After preparing gradient dilutions, the two yeast strains containing *Q*-*BD* + *AD-SPA* and *Q^c5^*-*BD* + *AD*-*SPA* were cultured on double (SD/−Leu/−Try) and quadruple (SD/−Leu/−Try/−His/−Ade + X-α-Gal) dropout SD media (Clontech) in plates incubated at 28 °C for 3 days.

To determine whether Q can bind to the promoters of *SSP* genes, yeast one-hybrid assays were performed according to the Matchmaker Gold Yeast One-Hybrid Library screening user manual (Clontech). The HMW-GS (Bx7, Dx5, and Dy10) and LMW-GS (1D1) gene promoters were used as the bait sequences. The prey sequence (*Q*) was cloned into the pGADT7-Rec vector and then inserted into the bait yeast strains. To examine the potential interaction between Q and the *SSP* gene promoters, the bait yeast strains were grown on medium lacking uracil but supplemented with different aureobasidin A concentrations. The yeast cells were subsequently added to medium lacking leucine and the resulting colonies were photographed. The primers used are listed in Table S7.

### 4.10. Dual-Luciferase Reporter Assay

A dual-luciferase reporter assay was performed as described by Song [82], with some modifications. The *SPA*, *Q* allele, and *Q^c5^* allele CDSs were inserted into the pGreenII 62-SK vector [83] under the control of the cauliflower mosaic virus 35S promoter (*35S:SPA*, *35S:Q*, and *35S:Q^c5^*). The *LMW*-*1D1* promoter was incorporated into the pGreenII 0800-*LUC* (firefly luciferase) vector, which contains the *35S:REN* (*Renilla reniformis* luciferase) cassette [83], to produce the reporter vector *LMW*-*1D1pro:LUC*. The primers used are listed in Table S7. The constructs were separately inserted into *A. tumefaciens* strain GV3101 cells (Weidi Biotechnology, Shanghai, China) along with the helper plasmid pSoup-19. The transformed *A. tumefaciens* cells were used in the following assay. Briefly, individual colonies carrying the above constructs were grown in YMB (yeast mannitol medium) liquid medium containing appropriate antibiotics, harvested, and resuspended in infiltration solution (10 mM MgCl_2_, 10 mM 4-morpholineethanesulfonic acid hydrate, and 150 mM acetosyringone) for an OD_600_ of 0.2. The cell suspensions were mixed (1:1 volume or a specific ratio) and injected into *N. benthamiana* leaves. The leaves co-infiltrated with *LMW*-*1D1pro:LUC* and the empty pGreenII 62-SK vector served as controls. All infiltrations were repeated at least three times, with at least three leaves per infiltration.

Leaf samples were collected using a hole punch at 48–60 h post-infiltration and then the LUC and REN activities were measured using the Dual Luciferase Reporter Assay Kit (Vazyme) and the GloMax^®^ 96 Microplate Luminometer (Promega, Madison, WI, USA) as previously described [83]. The LUC-to-REN activity (LUC/REN) ratio was used to estimate the transactivation by the effectors. Images of fluorescence were captured using the ChemiDoc™ Touch Imaging System (Bio-Rad, Hercules, CA, USA). Three biological replicates were examined per experiment.

### 4.11. Statistical Analysis

All data were compiled using Excel 2016 (Microsoft 2016, Redmond, WA, USA) and analyzed using the Data Processing System software (version 17.10) [84]. A one-tailed Student’s *t*-test was used for comparing two groups. For multiple comparisons, the Student’s *t*-test and an analysis of variance were performed (least significant difference). Data were recorded as the mean ± standard deviation.

## 5. Conclusions

The present study verified the positive effect of *Q* on GPC and the bread-making quality of wheat on the basis of an analysis of a new *Q* allele (*Q^c5^*). A missense mutation outside of the *Q^c5^* miRNA172-binding site leads to increased expression because of the associated highly folded mRNA secondary structure around the miRNA172-binding site, ultimately leading to an increase in spike density. Additionally, the missense mutation in *Q^c5^* increases GPC and the bread-making quality of wheat by repressing the expression of *SPR* and modulating the interaction between Q and SPA.

## Figures and Tables

**Table 1:** Expression of the genes encoding transcription factors that regulate *SSP* gene expression.

Gene ID	Log_2_FC	Gene Name
TraesCSU02G137200	1.42	*SPR-A* [22]
TraesCS2B02G616900	−3.89	*SPR-B* [22]
TraesCS2D02G567000	−8.22	*SPR-D* [22]
TraesCS3A02G077900	−0.31	*TaNAC019-A* [23]
TraesCS3B02G092800	−0.0037	*TaNAC019-B* [23]
TraesCS3D02G078500	0.996	*TaNAC019-D* [23]
TraesCS5A02G440400	0.32	*TaSHP* [26]
TraesCS5B02G444100	0.46	*TaSHP* [26]
TraesCS5D02G447500	0.34	*TaSHP* [26]
TraesCS1B02G343500	0.13	*SPA-B* [14,24]
TraesCS1D02G332200	0.35	*SPA-D* [14,24]
TraesCS3A02G336500	−0.61	*TaGAMYB-A* [20]
TraesCS3B02G367500	0.58	*TaGAMYB-B* [20]
TraesCS3D02G329400	0.49	*TaGAMYB-D* [20]
TraesCS3A02G249100	−0.07	*TaFUSCA3* [19]
TraesCS3B02G278000	−0.38	*TaFUSCA3* [19]
TraesCS3D02G249100	0.07	*TaFUSCA3* [19]
TraesCS5A02G155900	0.47	*TaPBF*
TraesCS5B02G154100	0.49	*TaPBF*
TraesCS5D02G161000	0.14	*TaPBF*
TraesCS7A02G205100	−0.23	*TaODORANT1* [21]
TraesCS7B02G112400	0.12	*TaODORANT1* [21]
TraesCS7D02G208000	−0.04	*TaODORANT1* [21]

## Data Availability

All data supporting the findings of this study are available within this article and the Supplementary Materials published online.

## Supplementary Materials

The following supporting information can be downloaded at: https://www.mdpi.com/article/10.3390/ijms23147581/s1. Reference [85] is cited in the supplementary figures.
