# Supplementary material for: The Qc5 Allele Increases Wheat Bread-Making Quality by Regulating SPA and SPR
Source: Int J Mol Sci. 2022 Jul 8;23(14):7581. doi: 10.3390/ijms23147581 (PMC9323144; doi:10.3390/ijms23147581)
Supplement: Supplementary file 1 [file ijms-23-07581-s001.zip › Supplementary figures.pdf]

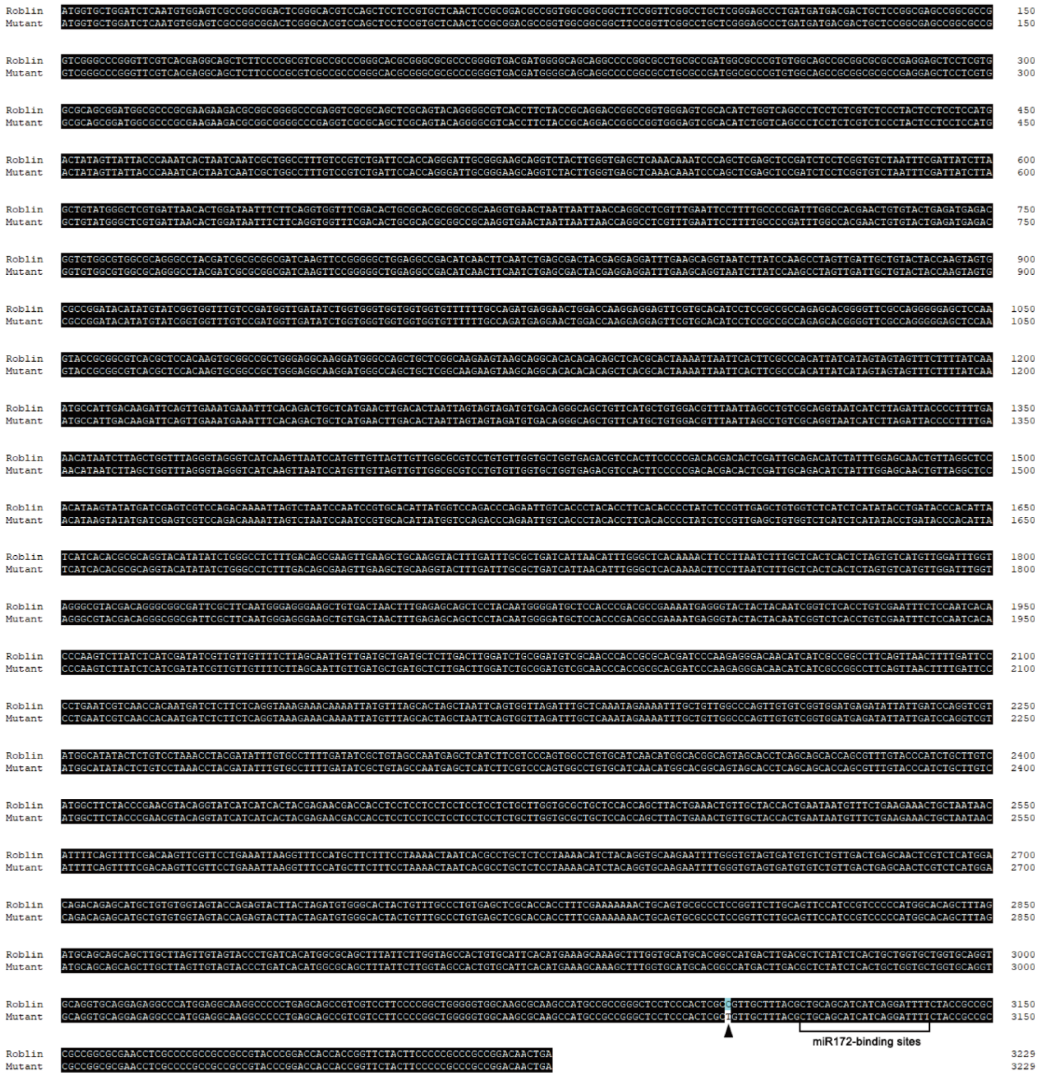

Figure S1. Alignment of the DNA sequences of *Q* and Q^{c5} alleles. The miRNA172-binding site is boxed with black lines, and the SNP flanking the miRNA172-binding site is indicated with black arrow.

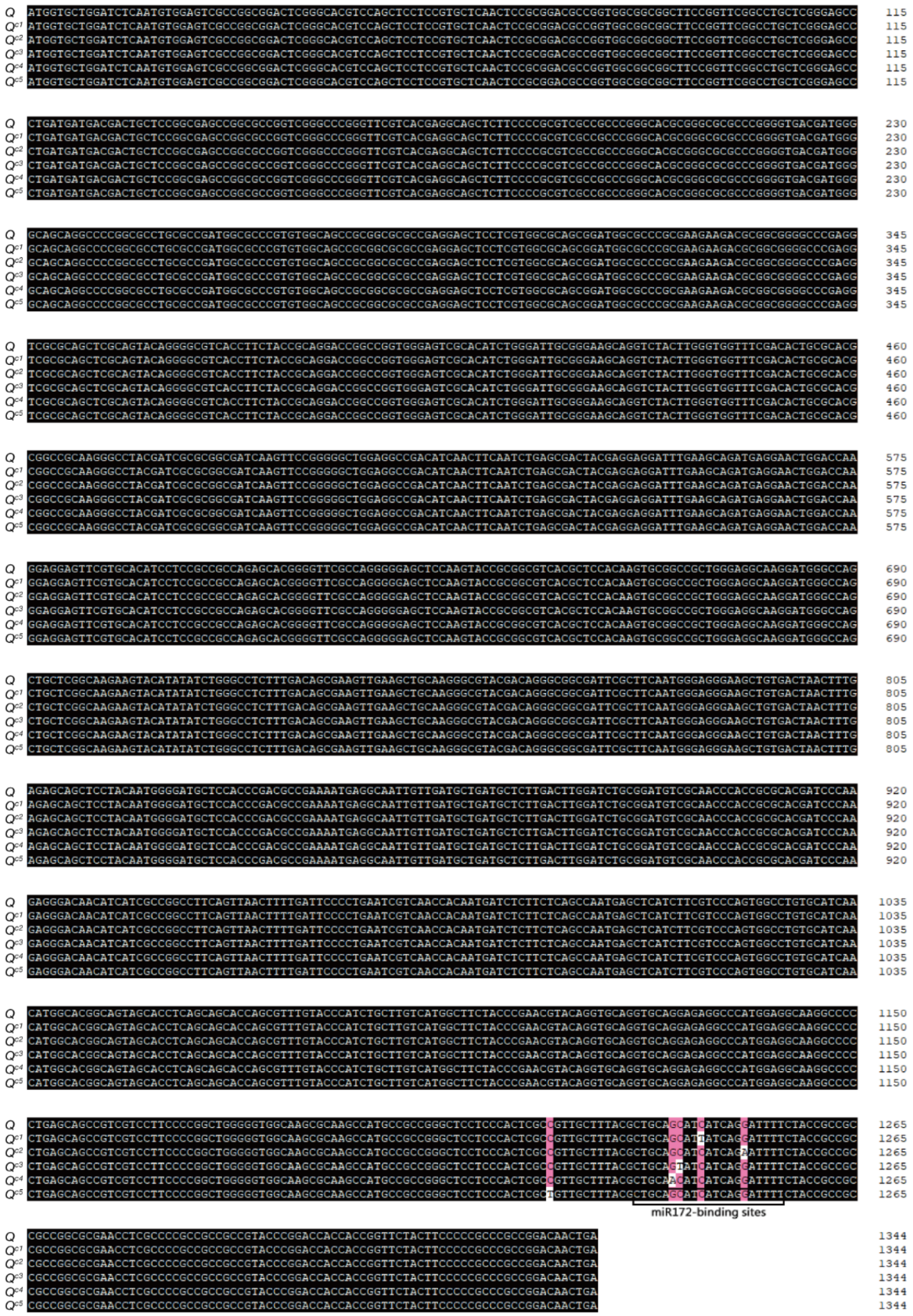

Figure S2. Alignment of the cDNA sequences of *Q* and five Q^c alleles. The miRNA172-binding site is boxed with black lines.

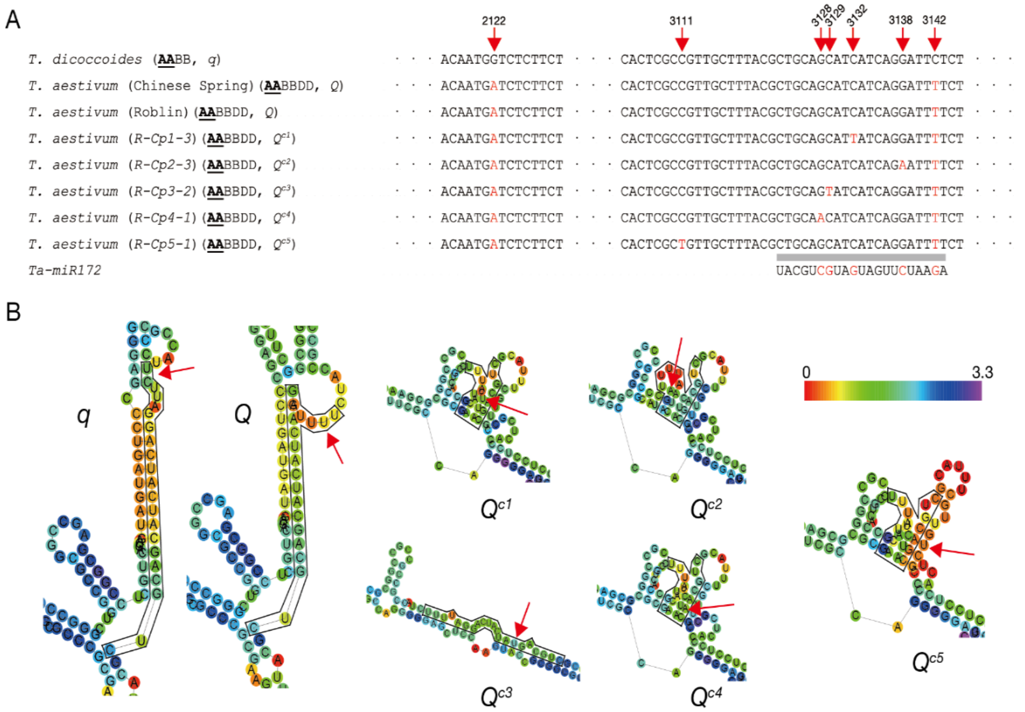

Figure S3. Effect of point mutations on the RNA secondary structure around the miRNA172-binding site. (A) Seven point-mutations in the DNA sequences of $q/Q/Q^{c1}/Q^{c2}/Q^{c3}/Q^{c4}/Q^{c5}$ alleles. The underlined is the miRNA172-binding site. The Genebank numbers are in order of AY702957.1, KX620763.1, KX620765-KX620768, and MW419115. (B) Comparison of the predicted RNA secondary structures of $q/Q/Q^{c1}/Q^{c2}/Q^{c3}/Q^{c4}/Q^{c5}$ alleles around the miRNA172-binding site, which drawing encoding positional entropy. The heat maps indicate the positional entropy, from low (red) to high (purple). The miRNA172-binding site is boxed with black lines. The red arrows represent the point mutations.

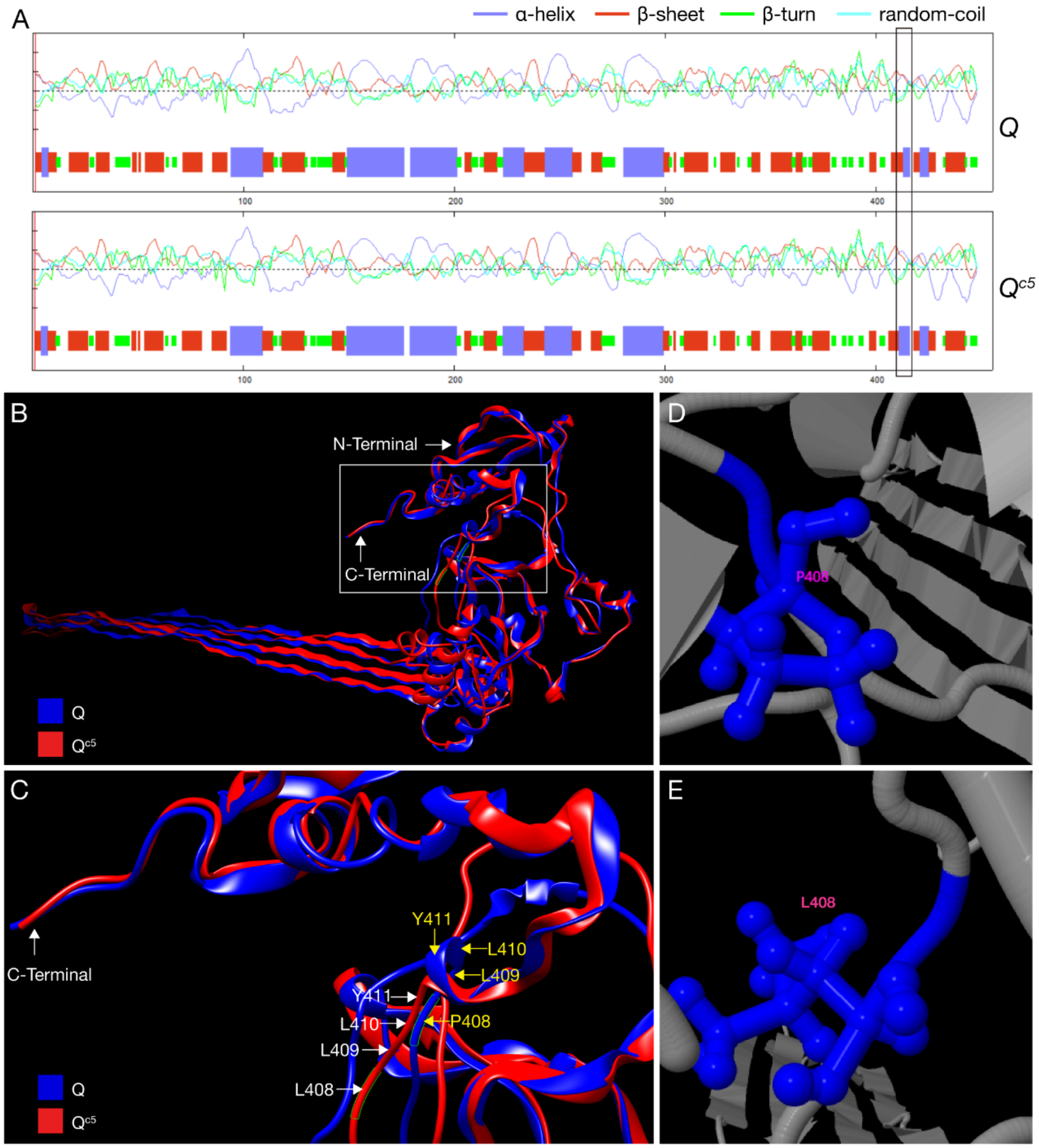

Figure S4. The number of amino acid residues for forming α-helix is more in Q^{c5} than in Q around the changed residue. (A) Comparison of predicted secondary structures of Q and Q^{c5}. The difference between Q and Q^{c5} is boxed by black line. (B-E) Comparison of 3D structure model of Q and Q^{c5}. (B) The superposition of full-length Q and Q^{c5} was compared by TM-align. (C) Magnification of the white box in (B). The yellow arrows show the position and type of amino acid residues in Q and the white arrows show that in Q^{c5}. The α-helix in the is ended in 409th residue of Q protein and 411th residue in Q^{c5}. (D) and (E) show the structure of 408th residue, in which (D) present proline in Q and (E) present leucine in Q^{c5}.

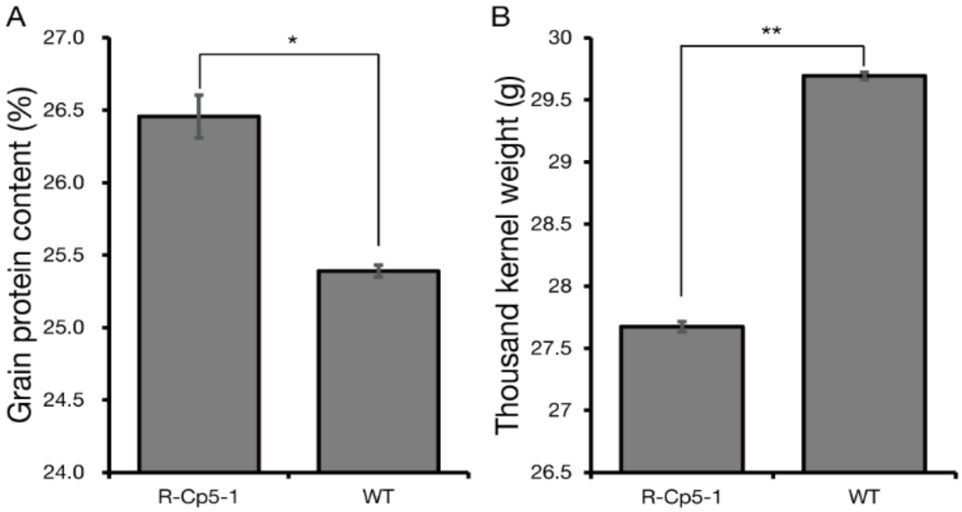

Figure S5. Comparison of the grain protein content (A) and thousand kernel weight (B) between *R-Cp5-1* and its WT in the glasshouse experiment.

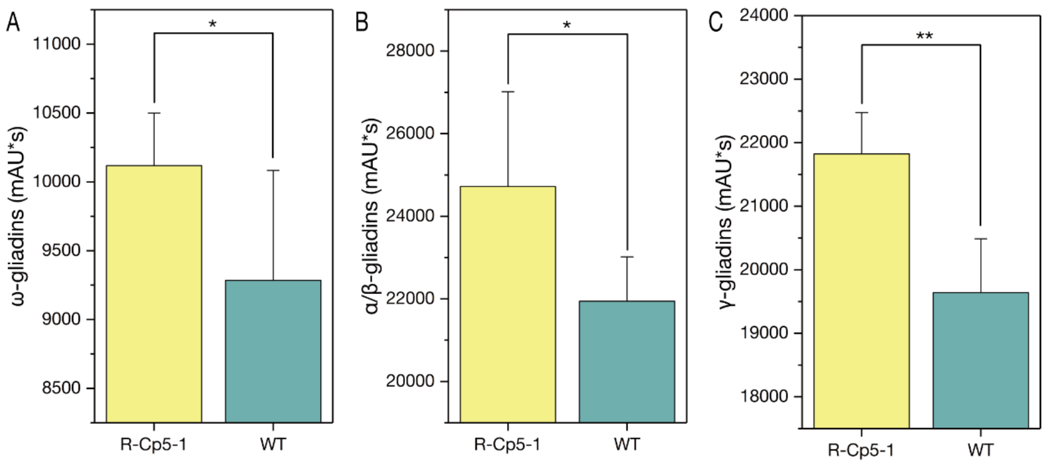

Figure S6. Comparison of the contents of α- (α/β-) (A), γ- (B) and ω-gliadins (C) of *R-Cp5-1* and its WT.

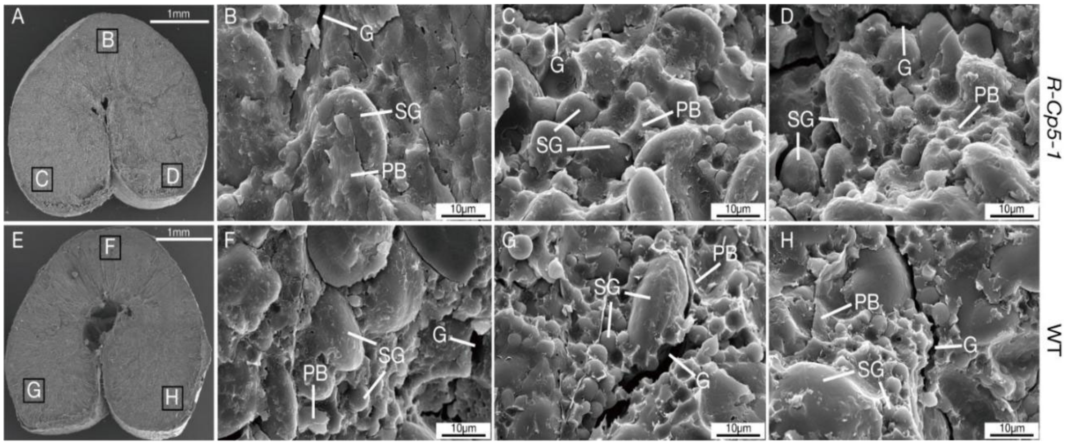

Figure S7. Scanning electron microscopic observation of three representative areas of the endosperm of mature seeds. (A-D) and (E-H) are images of cross-sections of *R-Cp5-1* and its WT, respectively. (B-D, E-H) Magnifications of the black boxes in (A) and (E), respectively. PB, protein body; SG, starch granule; G, gap between PB and SG. Scale bars, 1 mm in (A) and (E), and 10 μm in others.

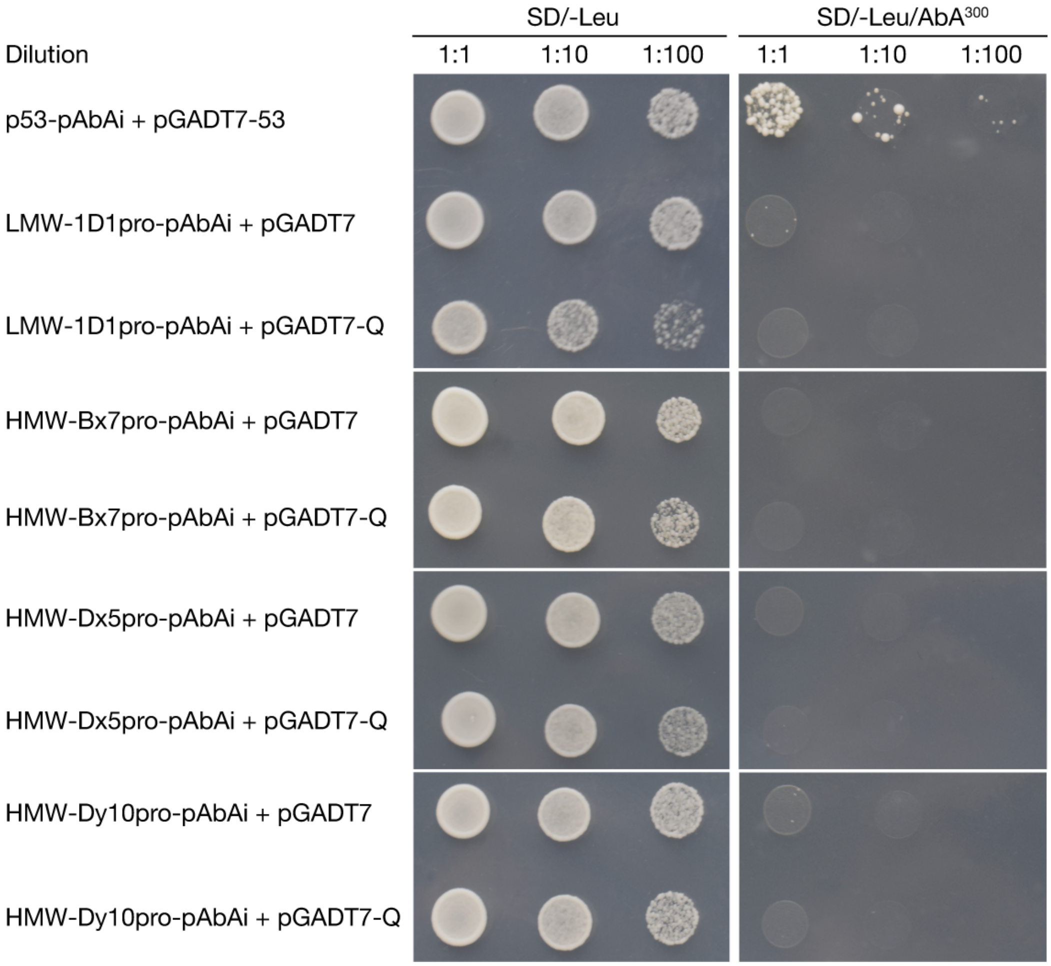

Figure S8. Yeast one-hybrid analysis showing that Q cannot bind the promoter of SSPs. Yeast cells were co-transformed with each combination. Cells were grown in liquid medium to an OD_{600} of 0.7, and a dilution series was prepared. For each dilution, cells were spotted onto medium (synthetic dropout, -Leu) supplemented with 300 ng/mL Aureobasidin A (AbA) to suppress background growth. The combination of p53-pAbAi and pGADT7-53 was used as positive control; each SSP promoter was co-transformed with pGADT7 as its negative control.

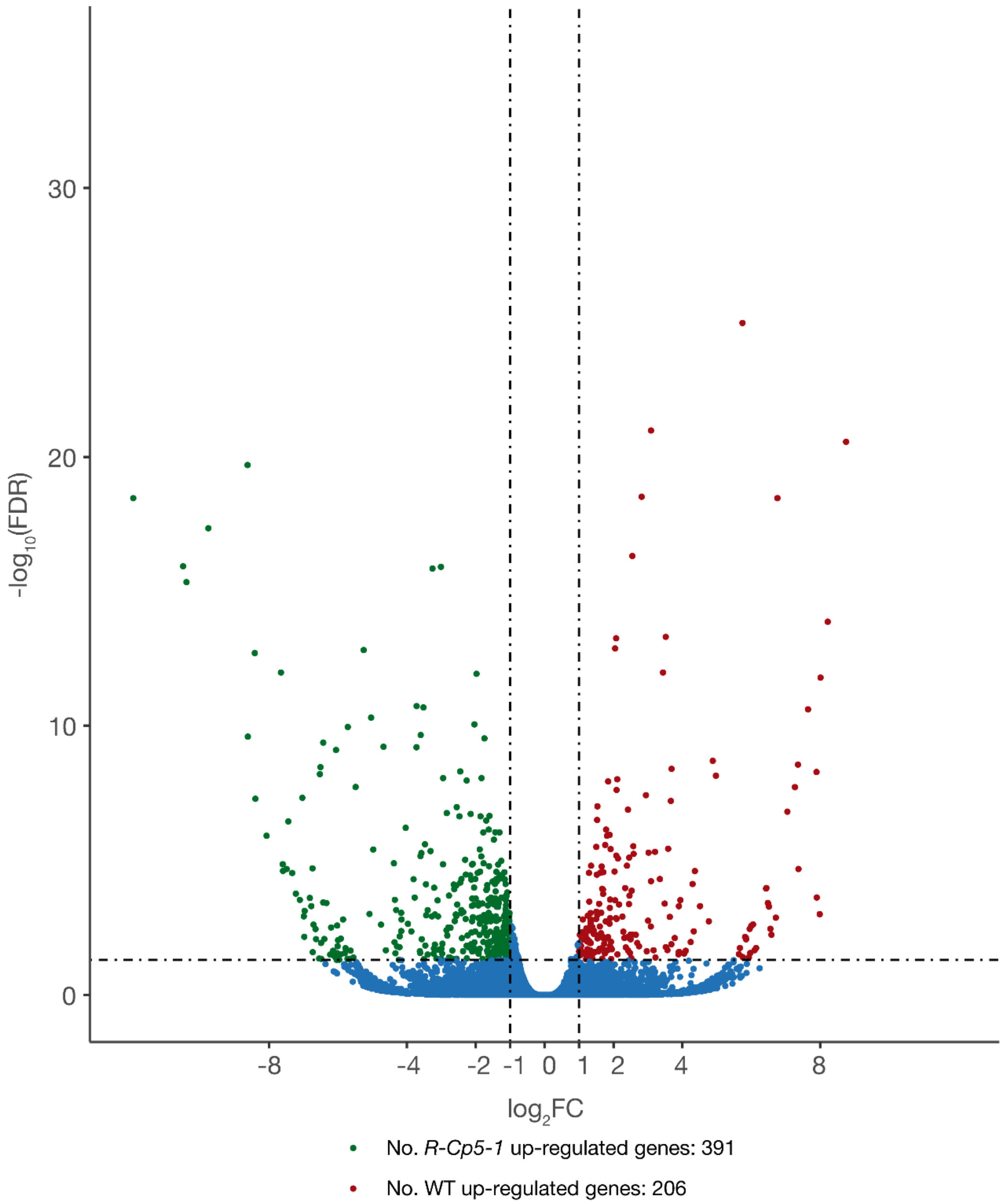

Figure S9. Volcano plot shows the DEGs in RNA-seq data.

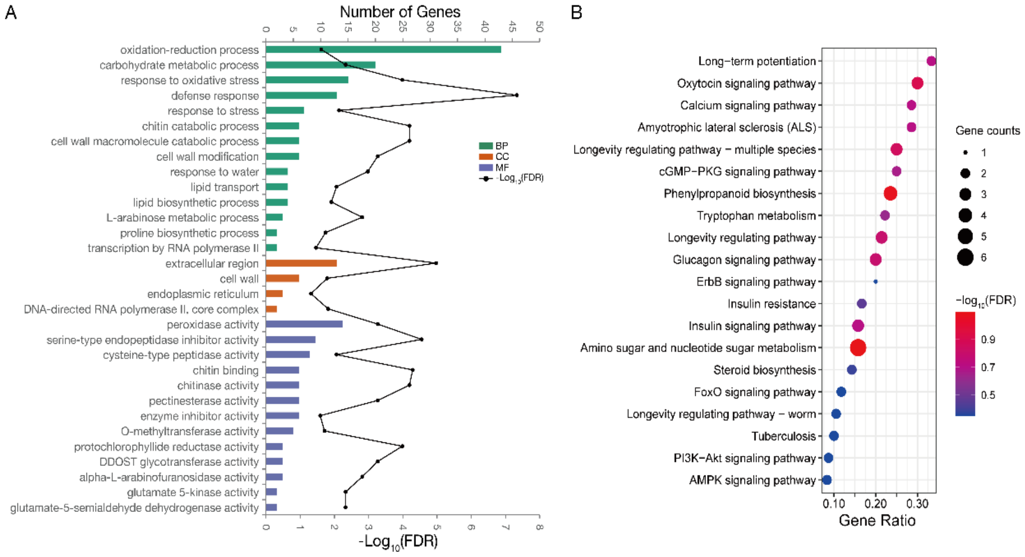

Figure S10. GO items (A) and top 20 KEGG pathways (B) enriched in DEGs between *R-Cp5-1* and its WT.

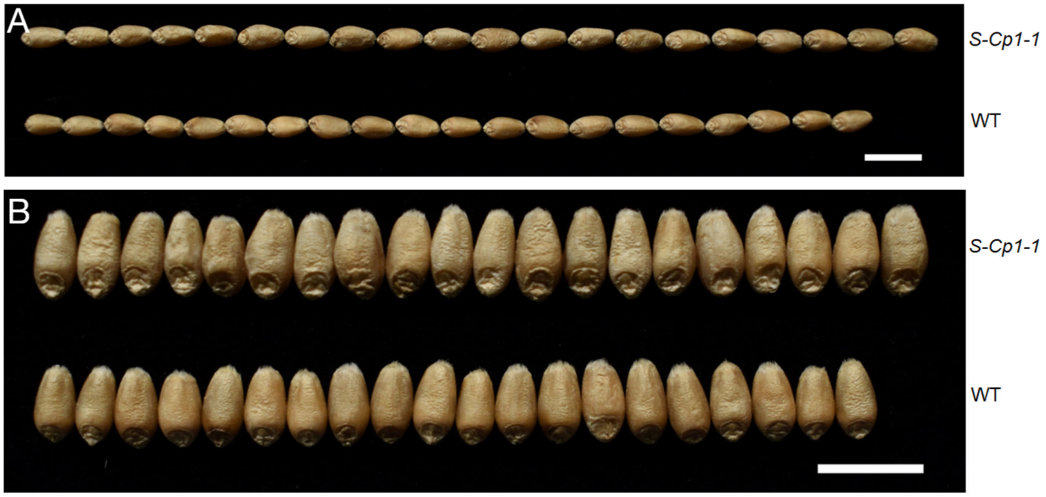

Figure S11. Comparison of seed length and seed width between *S-Cp1-1* (with Q^{c1} allele) and its WT (with *Q* allele). Length (A) and width (B) of 20 seeds of *S-Cp1-1* (up) and its WT (down). Scale bar, 1 cm.

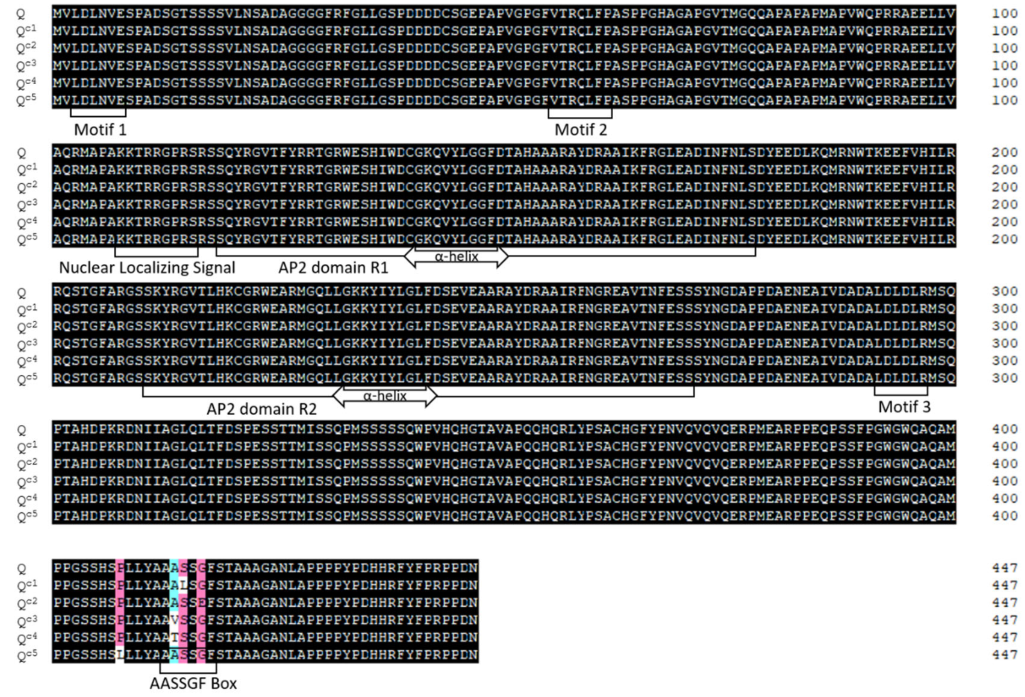

Figure S12. Alignment of the deduced amino acid sequences of *Q* and five *Q*^{c} alleles. Distribution pattern of the conserved domains was taken from Gil-Humanes [1].

1. Gil-Humanes, J.; Pistón, F.; Martín, A.; Barro, F. Comparative genomic analysis and expression of the *APETALA2*-like genes from barley, wheat, and barley-wheat amphiploids. *BMC Plant Biol* **2009**, 9, 66.

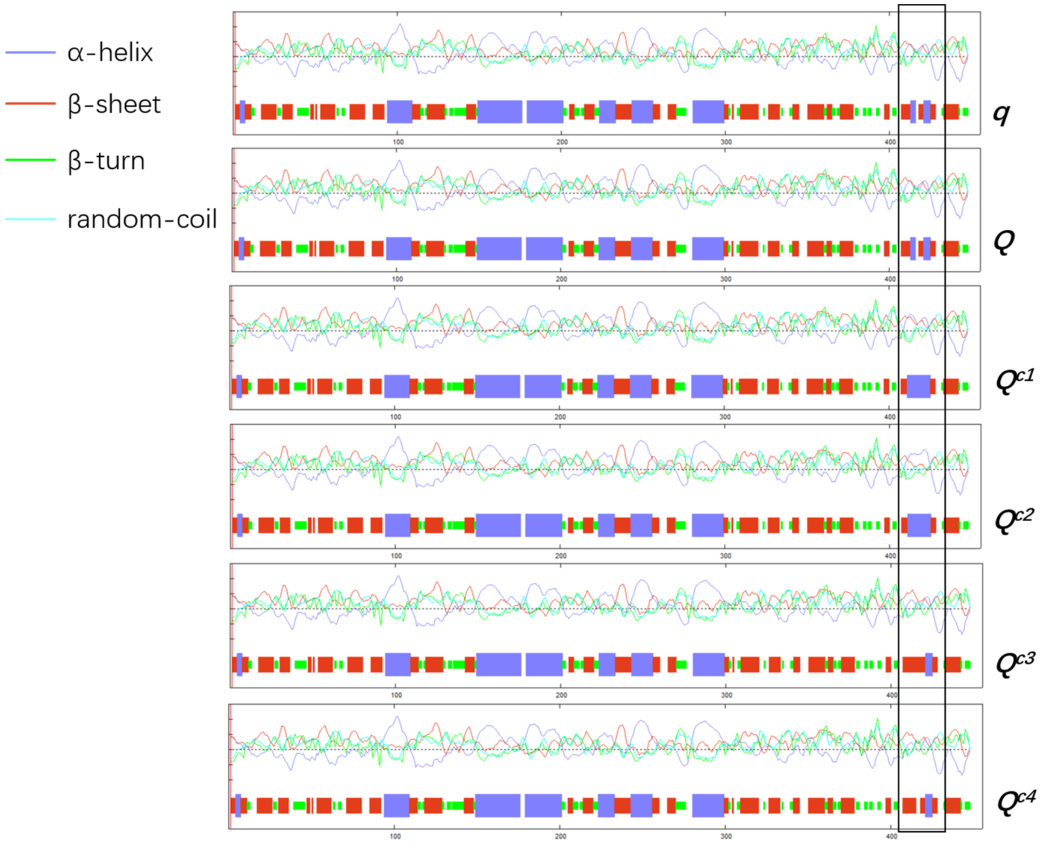

Figure S13. Comparison of the predicted secondary structures of *q*, *Q*, and Q^{c1}-Q^{c4}. The black frame highlights the amino acid substitutions.

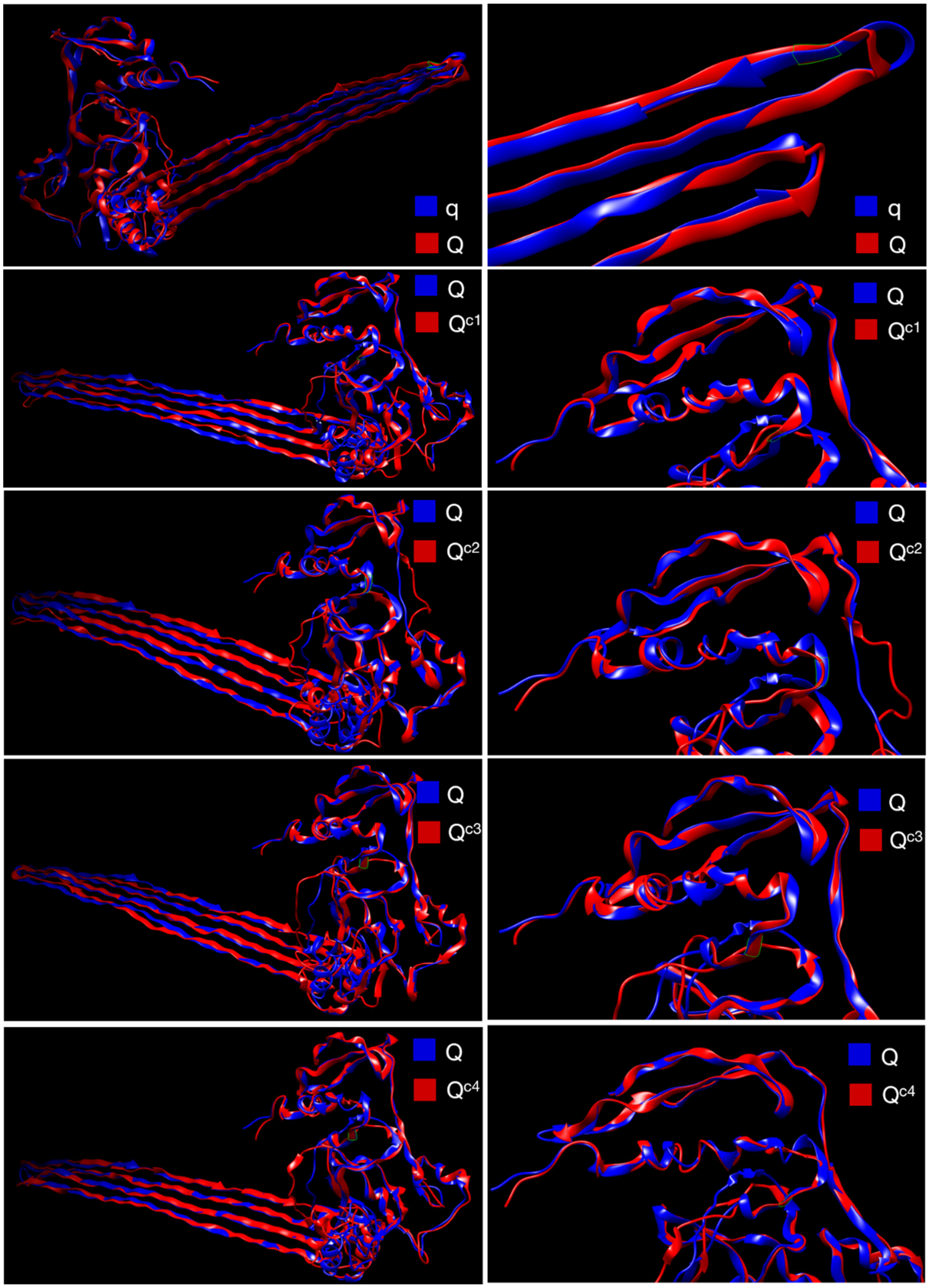

Figure S14. Comparison of the predicted 3D protein structures of q, Q, and Q^{c1}-Q^{c4}.
